# Brown adipose tissue ameliorates autoimmune arthritis via inhibition of Th17 cells

**DOI:** 10.1038/s41598-020-68749-x

**Published:** 2020-07-23

**Authors:** Jeonghyeon Moon, Dasom Kim, Eun Kyung Kim, Seon-yeong Lee, Hyun Sik Na, Gyoung Nyun Kim, Aram Lee, KyungAh Jung, Jeong Won Choi, Sung-Hwan Park, Sangho Roh, Mi-La Cho

**Affiliations:** 1 Laboratory of Immune Network, Conversant Research Consortium in Immunologic Disease, College of Medicine, The Catholic University of Korea, Seoul, 06591 Republic of Korea; 2 Rheumatism Research Center, Catholic Research Institute of Medical Science, College of Medicine, The Catholic University of Korea, 222 Banpo-Daero, Seocho-gu, Seoul, Republic of Korea; 3 Impact Biotech, Korea 505 Banpo-dong, Seocho-ku, Seoul, 137-040 Republic of Korea; 4 Division of Rheumatology, Department of Internal Medicine, Seoul St. Mary’s Hospital, College of Medicine, The Catholic University of Korea, Seoul, 06591 Republic of Korea; 5 College of Medicine, The Catholic University of Korea, Seoul, 06591 Republic of Korea; 6 Cellular Reprogramming and Embryo Biotechnology Laboratory, Dental Research Institute, BK21 PLUS Dental Life Science, Seoul National University School of Dentistry, Seoul, 08826 Republic of Korea; 7 Department of Medical Lifescience, College of Medicine, The Catholic University of Korea, 222, Banpo-daero, Seocho-gu, Seoul, 06591 Republic of Korea

**Keywords:** Cell biology, Immunology, Molecular biology, Rheumatology

## Abstract

The functions of adipose tissue are associated with autoimmune diseases, such as rheumatoid arthritis (RA). Some studies have shown that the three compositions of adipose tissue (white, brown, and beige) have different functions. Brown adipose tissue (BAT) is known to secrete several factors that differ from those in white adipose tissue. This suggests that BAT might have potential positive advantages in the physiology of autoimmune diseases. We compared the functions of collagen-induced arthritis mice-derived BAT (CIA BAT) with normal mice-derived BAT. DBA/1J mice (6–7 weeks of age) were immunized by intradermal injection at the base of the tail with 100 μg of bovine type II collagen (CII) emulsified in complete Freund’s adjuvant. Immunized mice then received booster immunizations by intraperitoneal injection with 100 μg of CII in incomplete Freund’s adjuvant. We transplanted CIA BAT and normal BAT into CIA recipient mice. After transplantation, we measured the functions of CIA BAT and normal BAT in mice. Normal BAT-transplanted mice showed significantly lower scores of bone damage, inflammation, and cartilage damage. The proinflammatory cytokines in normal BAT-transplanted mice, such as IL-12, IL-17, IL-6, and tumor necrosis factor-α (TNF-α), tended to decrease. Microarray analysis showed that the PI3K-AKT signaling pathway and IL-17 levels of CIA BAT tissues were significantly higher than those of normal BAT tissues. These results suggest that the transplantation of normal brown fat may have a therapeutic effect in RA patients.

## Introduction

Adipose tissue, once regarded solely as a tissue that stores fat and serves a protective role, is now recognized as an organ with many significant physiological functions^1^, such as inflammation and tissue repair^2^, and endocrine functions^3^. It is well known that obese people and animals have high levels of proinflammatory cytokines, such as serum tumor necrosis factor-alpha (TNF-α), interleukin-1 beta (IL)-1β, IL-6, and IL-17 all of which are produced by macrophages derived from adipose tissue^4,5^. Adipose tissue constitutes the major source of cytokines, chemokines, and metabolically active mediators known as adipokines^6,7^. It has been suggested that inflammation, which is induced by adipose tissue, is associated with osteoarthritis^8–10^, rheumatoid arthritis (RA)^11–13^, type 2 diabetes mellitus^14–17^, and coronary artery disease^18,19^.

RA is linked with most components of metabolic syndromes, such as changes in body weight, dyslipidemia, adipokine resistance, and insulin resistance^20^. Some studies have shown that obesity elevates the expression levels of proinflammatory cytokines and reduces the expression levels of anti-inflammatory cytokines^21,22^. Notably, our prior studies involved the use of metformin for the regulation of obesity-associated autoimmune disease^23^ and the balance of white/brown adipose tissue in obese mice^24^.

Recent studies have shown that adipose tissue is composed of three types of tissue: white, brown, and beige^25^. However, because beige fat derives from white adipose tissue (WAT), through a process known as browning of WAT^26^, adipose tissue is generally classified as white or brown^27^. WAT is an energy reservoir for other organs that is capable of adjusting its storage and secretory role to the nutritional state of the organism^28^. The size and quantity of WAT increase to accommodate excess energy when the energy intake exceeds the energy expenditure^29^. Furthermore, WAT releases heat shock protein (Hsp72), macrophage chemoattractant protein (MCP-1), IL-6, and IL-10 when it becomes stressed^30^. In contrast, brown adipose tissue (BAT) is a heat-producing adipose tissue, a specialized organ responsible for thermogenesis, which is primarily found in infants; its abundance decreases with age, such that it is lost in adulthood^31,32^. Unlike WAT, BAT is equipped with abundant mitochondria and uniquely expresses uncoupling protein (UCP)-1^33^, which allows protons produced by lipolysis and glycolysis to reenter the mitochondrial matrix, while producing heat instead of adenosine triphosphate^34^. In addition, BAT is highly innervated by the sympathetic nervous system^35^ and contains developed vasculature^36^. BAT is known to secrete adiponectin and IL-6, similar to WAT^37^. However, several factors may be secreted by BAT alone, such as fibroblast growth factor 21 (FGF-21)^38^. FGF-21 is expected to play a vital role in anti-inflammation by down-regulating mRNA expression of IL-17, TNF-α, IL-1β, IL-6^39^. Taken together, endocrine factors produced by BAT might have beneficial effects in autoimmune diseases.

We hypothesized that BAT can be associated with treating RA by reducing inflammatory cytokines. IL-23-IL-17 axis is thought to have a critical role in RA^40^. Therefore, we thought that by reducing IL-17, further deterioration of RA could be prevented. Hence, we compared the functions of collagen-induced arthritis (CIA) mice-derived BAT (CIA BAT) with normal mice-derived BAT in this study. We injected CIA mice with CIA BAT or normal BAT and evaluated the therapeutic role of normal BAT in CIA mice.

## Results

### Experimental RA requires normal BAT activity for therapy

**Figure 1:**
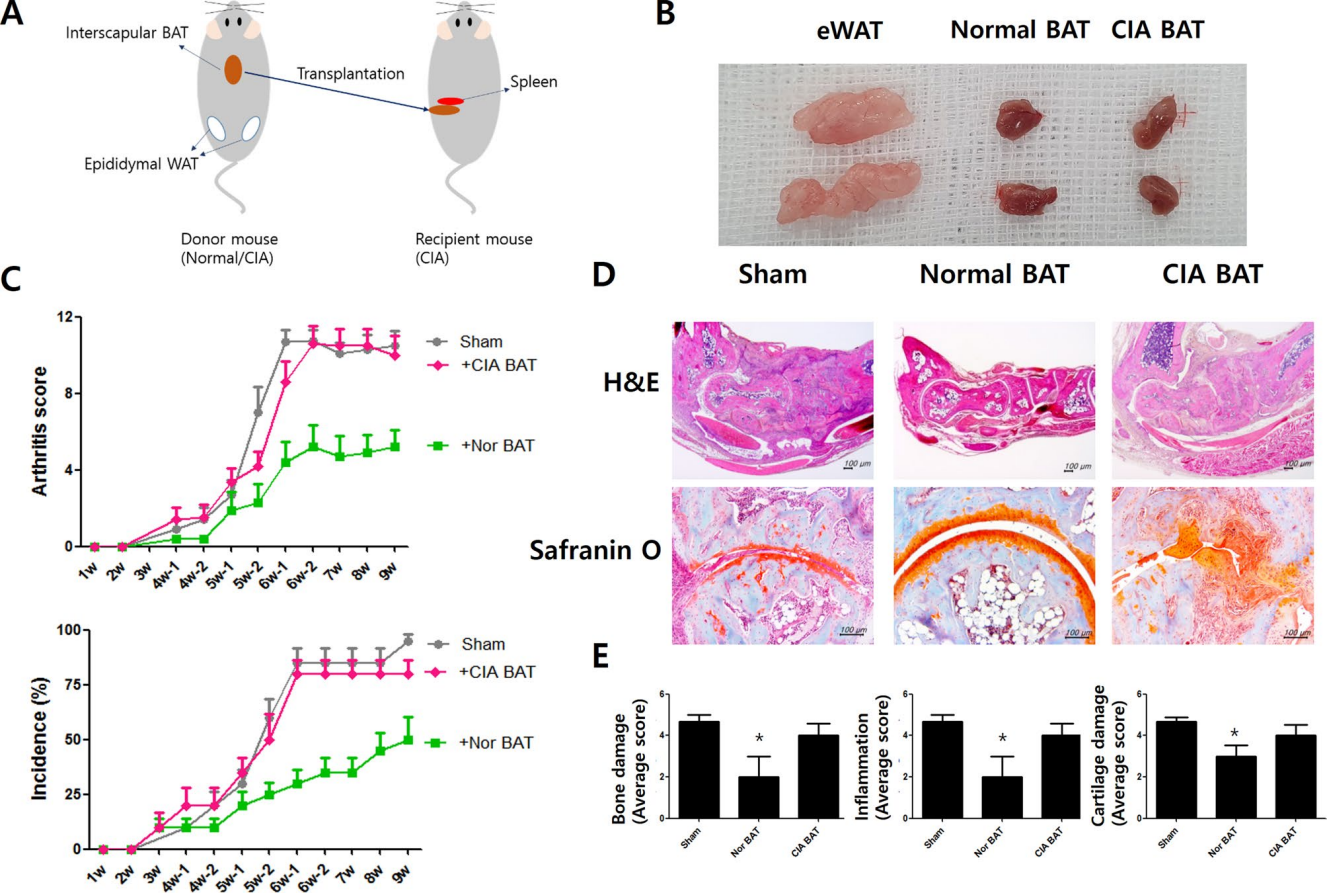
Transplantation of normal brown adipose tissue reduces tissue damage, inflammation, and development of experimental rheumatoid arthritis. (**A**) Schematic representation of the experiments. (**B**) Epididymal white adipose tissues (eWAT), normal brown adipose tissues (normal BAT) and collagen-induced arthritis mice-derived BAT (CIA BAT) were presented. (**C**) Reductions of arthritis score and arthritis incidence were observed in normal BAT-transplanted CIA mice. (**D**) Histological features of the joints were stained with hematoxylin/eosin and Safranin O. (**E**) The histological score of bone damage, inflammation and cartilage damage were presented. *P < 0.05. Scale bars = 100 µm.

The scheme of the transplantation of BAT was presented (Fig. 1A). Epididymal white adipose tissues (eWAT), normal BAT which was acquired to control mouse and BAT which was derived from CIA mouse (CIA BAT) were showed (Fig. 1B). Compared to mice that received sham surgery or transplantation of CIA BAT, normal BAT transplantation mice had significantly lower arthritis scores and incidence of arthritis throughout the experimental period (Fig. 1C). The joint tissues were presented using H&E and safranin O staining for the disruption of tissues (Fig. 1D). Histological analysis showed that the average scores of bone damage, inflammation, and cartilage damage of the normal BAT group were significantly lower than those of the control and CIA BAT groups (Fig. 1E).

### Transplantation of normal brown adipose tissue ameliorates proinflammatory cytokines and impacts anti-inflammatory cytokine secretion

**Figure 2:**
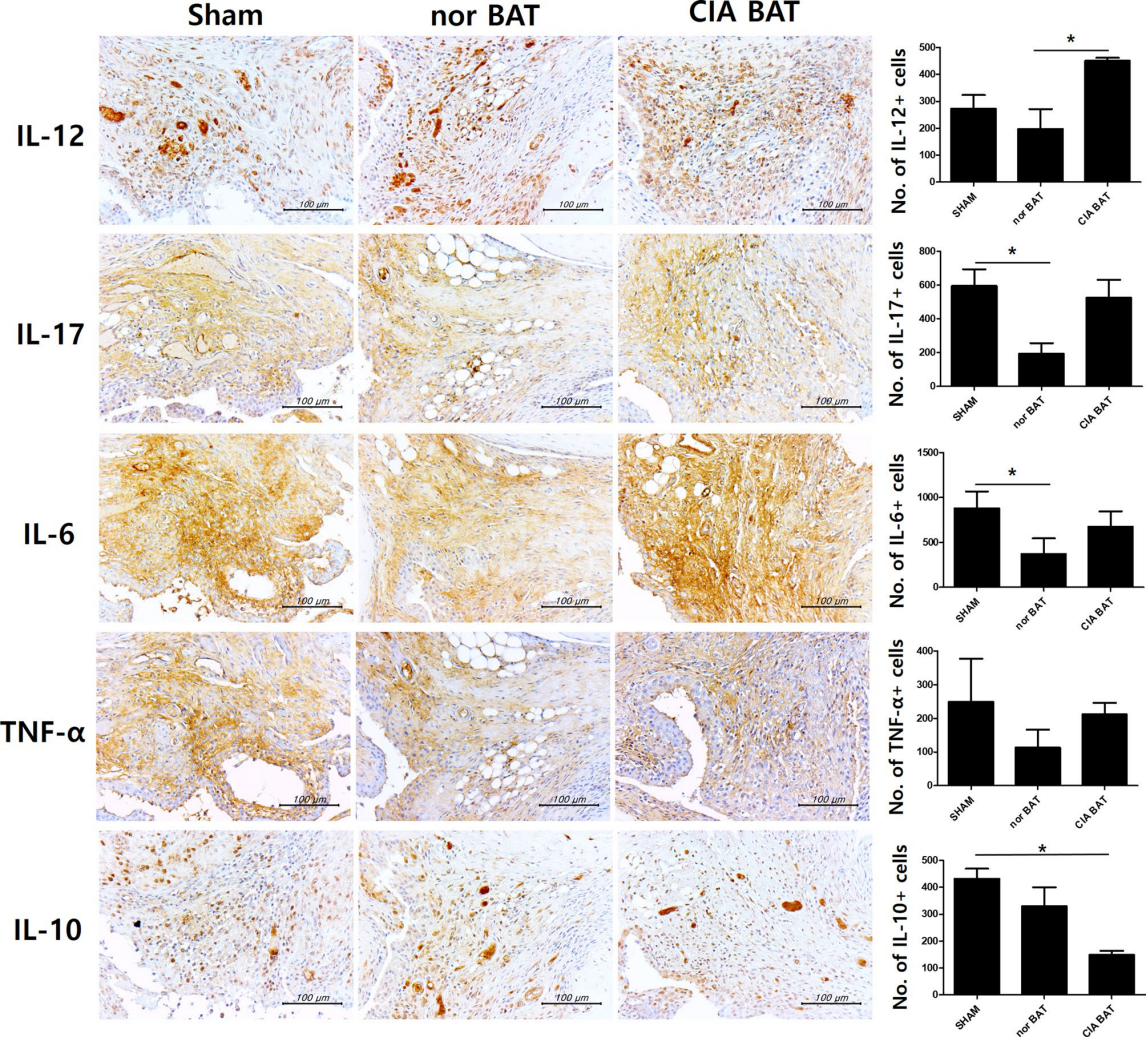
Transplantation of normal brown adipose tissue reduces secretion of proinflammatory cytokines. Immunohistochemistry images are shown with tissues stained by anti-IL-12, anti-IL-17, anti-IL-6, anti-TNF-α, and anti-IL-10 antibodies. *P < 0.05. Scale bars = 100 µm.

Inflammatory cytokines were observed by immunohistochemistry in murine joint tissues. Levels of proinflammatory cytokines, such as IL-12, IL-17, IL-6, and TNF-α, tended to decrease in normal BAT-transplanted mice. IL-12 significantly increased in CIA BAT-transplanted mice, compared to its levels in normal BAT-transplanted mice. Levels of IL-10, an anti-inflammatory cytokine, significantly differed between sham- and CIA BAT-transplanted mice. However, there were no significant differences between sham- and normal BAT-transplanted mice (Fig. 2).

### Normal BAT ameliorates balance of Th17/Treg cells

**Figure 3:**
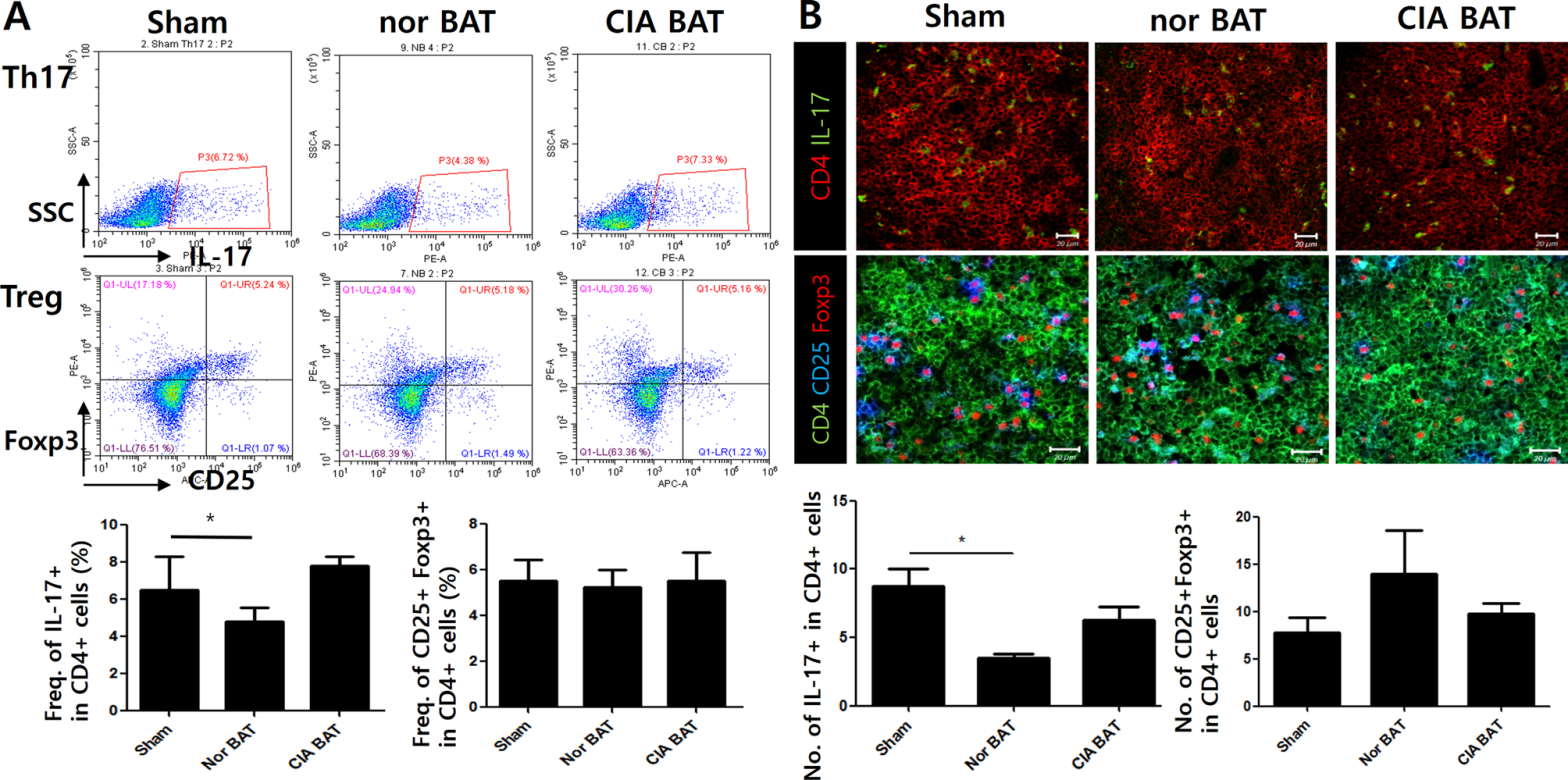
Populations of Th17 and Treg cells were reduced by transplantation of normal BAT in CIA mice. (**A**) Numbers of Th17 and Treg cells were measured by ex vivo FACS in the splenocytes of control and adipocyte tissue-transplanted mice. (**B**) Splenic tissue from each group of mice was monitored by immunofluorescence staining. *P < 0.05. Scale bars = 20 µm.

To quantify the populations of Th17 and Treg cells, splenocytes were analyzed by flow cytometry after mice had been sacrificed. The populations of Th17 and Treg cells did not significantly differ in each group. However, Th17 cells in the normal BAT group tended to be fewer than those in the control and CIA BAT groups (Fig. 3A). Confocal imaging showed significantly fewer CD4^+^IL-17^+^ cells in the spleen tissues of normal BAT-transplanted mice than in the spleen tissues of sham- or CIA BAT-transplanted mice. Although CD4^+^CD25^+^ Foxp3^+^ cells did not significantly differ in each group, CD4^+^CD25^+^ Foxp3^+^ cells showed a tendency to increase in the normal BAT-transplanted group, compared to the sham- or CIA BAT groups (Fig. 3B).

### Brown adipose tissue inhibits IL-17 secretion and strongly induces IL-10 expression

**Figure 4:**
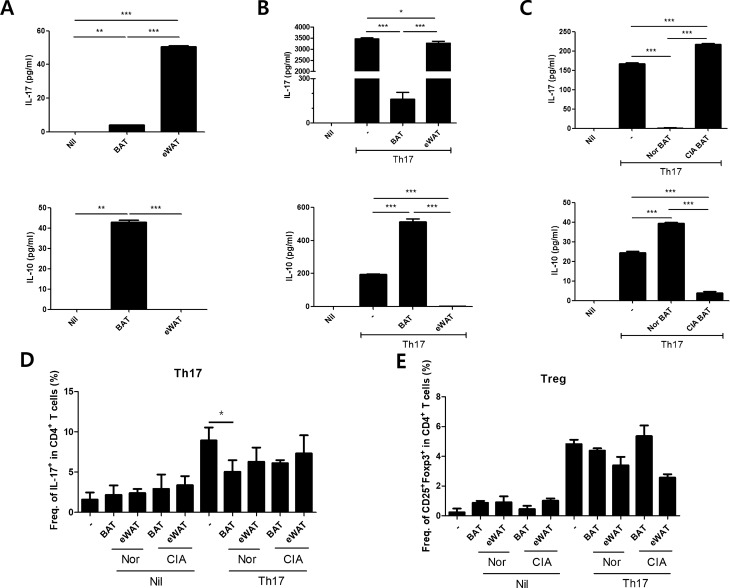
BAT inhibits Th17 differentiation and induces IL-10 expression in vitro. (**A**) IL-17 and IL-10 levels were measured by ELISA in BAT and eWAT. (**B**) Levels of IL-17 and IL-10 in BAT and eWAT were detected by ELISA under Th17-inducing conditions for 72 h. (**C**) IL-17 and IL-10 levels of normal BAT and CIA BAT were measured by ELISA. (**D**,**E**) The population of Th17 cells and Treg cells in BAT co-culture condition were measured. *P < 0.05, **P < 0.01, and ***P < 0.005.

To determine the expression of IL-17 and IL-10 in each adipose tissue, BAT, epididymal white adipose tissue (eWAT) and CIA BAT were cultured for 72 h. IL-17 and IL-10 levels were detected by ELISA in BAT culture medium and eWAT culture medium, respectively. In prevalent condition, IL-17 of brown fat tissues was lower expressed compared with eWAT, however, IL-10 which is anti-inflammatory cytokine were highly expressed in brown fat tissues. (Fig. 4A). Furthermore, IL-17 expression of eWAT was higher than BAT and IL-10 expression of eWAT was lower than the BAT in the Th17 differentiation condition (Fig. 4B). The level of IL-17 of CIA BAT was more elevated than normal BAT and the level of IL-10 was decreased in CIA BAT compared with normal BAT (Fig. 4C). To investigate BAT decreases the population of Th17, the differentiated-Th17 cells derived from CD4 positive splenocytes were cultured with BAT and eWAT respectively. Although the population of Treg cells showed no significant differences, the population of Th17 cells was decreased in BAT co-culture condition (Fig. 4D,E).

### Comparison of gene expression between normal BAT with CIA BAT in murine

**Figure 5:**
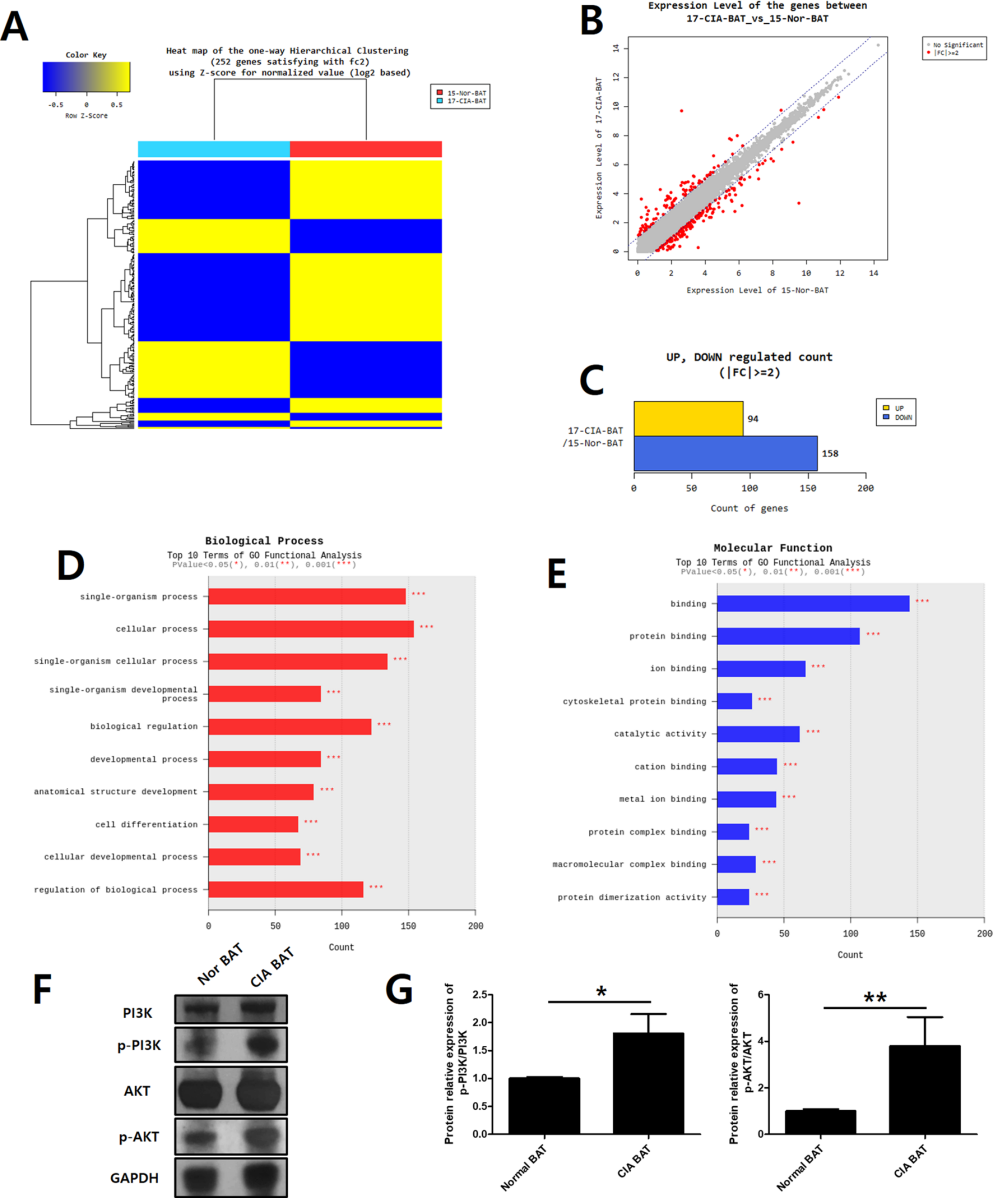
Comparison of gene expression between normal BAT and CIA BAT in mice by microarray analysis. (**A**) Hierarchical cluster heatmap of normal BAT and CIA BAT. (**B**) Scatter plots of the expression levels of genes between normal BAT and CIA BAT. (**C**) Significantly different gene numbers are shown in normal BAT and CIA BAT. (**D**,**E**) Top 10 biological processes and molecular functions associated with upregulated and downregulated genes in normal BAT and CIA BAT, based on gene ontology functional analysis. (**F**) The relative expression levels of total PI3K (PI3K), phosphorylated PI3K (p-PI3K), total AKT (AKT) and phosphorylated AKT (p-AKT) proteins were determined by western blotting. (**G**) The protein expression levels which were the ratio of the phosphorylated protein/total protein values of PI3K and AKT were monitored. *P < 0.05, **P < 0.01, and ***P < 0.005.

We compared normal mice-derived BAT with CIA mice-derived BAT using a microarray (Fig. 5A). Differentially expressed genes, identified using an absent/present (A/P) classification and ≥ 2-fold difference as cut-offs, constituted 252 of 24,532 genes (Fig. 5B): 94 were upregulated and 158 were downregulated (Fig. 5C). The 10 dominant gene ontology (GO) terms of biological processes are presented in Fig. 5D. The 10 dominant GO terms of molecular function are presented in Fig. 5E.

### PI3K-AKT signaling-associated genes and IL-17 signaling-associated genes were highly expressed in CIA BAT, compared to normal BAT

According to the Kyoto Encyclopedia of Genes and Genomes, the PI3K-AKT and IL-17 signaling pathways significantly differed between CIA BAT and normal BAT tissues (Table 1). In the PI3K-AKT pathway, Flt1, Pik3r1, Sk1, and Nr4a1 were highly expressed in CIA BAT. Western blotting showed the expressions of PI3K/AKT pathway proteins (Fig. 5F). The expressions of phosphorylated PI3K and AKT was increased in CIA BAT than normal BAT (Fig. 5G). However, Fn1, Col4a5, Col1a1, and Col1a2 (PIK3-AKT signaling-associated genes and focal adhesion molecules) were downregulated in CIA BAT. IL-17 signaling pathway-associated genes, such as Jun, S100a8, and S100a9, were highly expressed in CIA BAT.

**Table 1:** The significant pathways in CIA BAT compared to nor BAT (*P* < 0.05).

Map name	P-value	Genes	Fold change (CIA BAT/nor BAT)	Description
PI3K-AKT signaling pathway	6.04637E−06	Flt1	2.107880	FMS-like tyrosine kinase 1
		Pik3r1	3.895963	Phosphatidylinositol 3-kinase, regulatory subunit
		Sgk1	2.421112	Serum/glucocorticoid regulated kinase 1
		Nr4a1	4.452061	Nuclear receptor subfamily 4, group A, member 1
		Col4a5	− 2.121215	Collagen, type IV, alpha 5
		Fn1	− 2.389394	Fibronectin 1
		Col1a2	− 2.766700	Collagen, type I, alpha 2
		Col1a1	− 3.589281	Collagen, type I, alpha 1
IL-17 signaling pathway	0.026892276	Jun	2.187605	Jun proto-oncogene
		S100a8	3.755533	S100 calcium binding protein A8 (calgranulin A)
		S100a9	4.426975	S100 calcium binding protein A9 (calgranulin B)

## Discussion

The role of adipose tissue was once presumed to be the storage of fat and protection of the body^41^. However, recent studies have identified other functions of adipose tissue and its association with autoimmune diseases, such as osteoarthritis and RA^42^. Brown adipose tissue, a subtype of adipose tissue, is well known for its heat-producing role^43^. In addition to this role, brown adipose tissue may be beneficial for the treatment of autoimmune diseases.

To determine whether functions differed between normal BAT and CIA BAT, we transplanted normal BAT or CIA BAT into CIA mice. The results showed that normal BAT-transplanted mice had lower arthritis scores and incidence of arthritis than control mice or CIA BAT-transplanted mice. This was confirmed by histological analysis. Safranin O staining showed that the cartilage of normal BAT-transplanted mice was healthier than that of sham- or CIA BAT-transplanted mice. The scores of bone damage, inflammation, and cartilage damage were significantly lower in the normal BAT-transplanted group. Furthermore, compared with the severity of arthritis in the normal CIA model and the sham CIA model, there were no notable differences (data not shown).

The proinflammatory cytokines of normal BAT-transplanted mice tended to decrease relative to those of other experimental groups, as evidenced by immunohistochemistry analysis of mouse joint tissues. IL-12, a Th1 cell-stimulating factor^44^, was significantly higher in CIA BAT-transplanted mice than in normal BAT-transplanted mice. Conversely, the expression of IL-10 was significantly lower in CIA BAT-transplanted mice than in control mice. There were no significant changes between sham- and normal BAT-transplanted mice.

Ex vivo flow cytometry and immunofluorescence staining analyses showed that the populations of Foxp3^+^ Treg cells did not significantly differ among groups. However, the population of Th17 cells was significantly reduced in normal BAT-transplanted mice, as evidenced by immunofluorescence. Flow cytometry showed that Th17 cells of normal BAT-transplanted mice tended to decrease. Although there is some controversy over the function of IFNγ^45,46^, Th1 and Th17 cells are known to be involved in the development of arthritis in the CIA model^47,48^. In this study, Th1 cells were determined by flow cytometry in each mouse groups, there were no significant differences respectively (data not shown).

Subsequently, IL-17 and IL-10 expression levels in the cell culture medium were measured by ELISA. Notably, the IL-17 expression level in eWAT was significantly higher than that in normal BAT, while the IL-10 expression level in eWAT was significantly lower than that in normal BAT. In Th17-inducing conditions, the expression levels of IL-17 in eWAT and CIA BAT were significantly higher than in normal BAT. IL-10 was highly expressed by normal BAT, but not by eWAT and CIA BAT. Taken together, these findings suggested that CIA BAT was similar to eWAT and distinct from normal BAT.

To determine differences between CIA BAT and normal BAT, we performed microarray analysis, which identified 252 differentially expressed genes. The 10 dominant GO terms of biological processes were identified; seven of these 10 terms were related to the regulation of cellular and biological processes (Fig. 5D). In the Kyoto Encyclopedia of Genes and Genomes enrichment data, 11 of the top 20 enrichment terms were related to human disease (data not shown). These data showed that brown fat in the RA mouse model differs from brown fat in normal mice.

Eight of the 10 dominant GO terms of molecular function were related to protein binding and molecular binding (Fig. 5E). PI3K signaling pathway-associated genes (Flt1, Pik3r1, Sgk1, and Nr4a1) showed increased expression in CIA BAT. Also, we assessed the expression of PI3K and AKT proteins by western blotting. The activated forms of PI3K and AKT were known to be phosphorylated^49,50^. Also, the activated form of PI3K and AKT were known to be increase of inflammation through secreting of pro-inflammatory cytokines such as IL-1β, IL-6 TNFα^51,52^. This study showed that the activated form of PI3K and AKT was increased in BAT of CIA mice than normal. The increased PI3K/AKT signaling is thought to aggravate inflammation in the CIA model. Other genes showed reduced expression in CIA BAT, such as Fn1, Col4a5, Col1a1, and Col1a2. These were both PI3k signaling pathway-associated genes and focal adhesion-associated genes.

The expression of genes in the IL-17 signaling pathway was higher in CIA BAT than in normal BAT (Table 1). Notably, proinflammatory genes (Jun, S100a8, and S100a9) were increased in CIA BAT, compared to normal BAT; these are closely related to inflammation. Taken together, these data indicate that the brown fat of the RA model mouse activates the PI3K pathway and promote inflammatory conditions. However, the reduction of focal adhesion molecules in CIA BAT can reduce cell survival.

Our study showed that normal brown fat had a therapeutic effect on the RA mouse model. However, brown fat derived from CIA mice did not have a therapeutic effect on other CIA mice. Normal brown fat reduced the pathological score of arthritis in CIA mice and reduced the levels of proinflammatory cytokines in tissues. In particular, brown fat tissue showed significant differences in proinflammatory cytokine (IL-17) and an anti-inflammatory cytokine (IL-10), compared with epididymal white tissue. Microarray data showed that expression levels in some pathways significantly differed between normal BAT and CIA BAT. Taken together, the brown fat of RA mouse model showed a similar aspect with white adipose tissues. Since normal brown adipose tissue has the ability to regulate immunity, we suggested that the normal brown fat transplantation or the trans-differentiation of white fat to brown fat may have therapeutic effects in RA patients.

## Materials and methods

### Animals

Male DBA/1J mice (6–8 weeks of age) were purchased from Orient Bio (Seoul, Korea). All mice were reared under specific pathogen-free conditions. All experiments were approved by the Animal Research Ethics Committee of the Catholic University of Korea (permit number: CUMC: 2015-0128-01) and conducted in accordance with the guidelines of the National Institutes of Health.

### Type II collagen (CII) immunization and induction of CIA

DBA/1J mice were immunized by intradermal injection at the base of the tail with 100 μg of bovine type II collagen (CII) emulsified in complete Freund’s adjuvant (Arthrogen-CIA, Redmond, WA, USA) (1:1, w/v). Two weeks later, the mice received booster immunizations by intraperitoneal injection with 100 μg of bovine CII in incomplete Freund’s adjuvant (DIFCO, Detroit, MI, USA) (1:1, v/v).

### Assessment of arthritis

Mice were observed three times per week for the onset, duration, and severity of joint inflammation for 9 weeks after the primary immunization. The severity of arthritis in each paw was graded in accordance with an established scoring system, as follows: 0 = normal, 1 = mild swelling, 2 = moderately severe arthritis involving toes and ankle, 3 = severe arthritis involving an entire paw, and 4 = severe arthritis resulting in ankylosis and loss of the joint; the maximum cumulative score for each animal was 16^53^.

### Immunohistochemistry

Mouse joint tissues were fixed in 4% paraformaldehyde, decalcified in EDTA bone decalcifier, and embedded in paraffin. Seven-micrometer sections were prepared and stained with hematoxylin/eosin and Safranin O to detect proteoglycans. The sections were dewaxed using xylene and dehydrated using an alcohol gradient. Endogenous peroxidase activity was quenched with 3% H_2_O_2_ in methanol_._ Immunohistochemistry was performed using the Vectastain ABC kit (Vector Laboratories, Burlingame, CA, USA). Tissues were incubated with primary anti-IL-17, anti-IL-10, anti-IL-12, anti-IL-6 and TNF-α antibodies (all from R&D Systems, Minneapolis, MN, USA) overnight at 4 °C, followed by incubation with a biotinylated secondary linking antibody and streptavidin–peroxidase complex for 1 h. The final color product was developed using DAB chromogen (DAKO, Carpinteria, CA, USA). The sections were counterstained with hematoxylin and photographed with an Olympus photomicroscope (Tokyo, Japan).

### Histological assessment of arthritis

The hematoxylin/eosin-stained sections were scored for inflammation and cartilage damage. Inflammation was scored in accordance with established criteria^54^, as follows: score 0: no inflammation; score 1: slight thickening of lining layer or some infiltrating cells in subliming layer; score 2: slight thickening of lining layer and some infiltrating cells in subliming layer; score 3: thickening of lining layer, influx of cells in subliming layer, and presence of cells in the synovial space; and score 4: synovium highly infiltrated with many inflammatory cells. Cartilage erosion was scored as follows: score 0: no destruction; score 1: minimal erosion limited to single spots; score 2: slight to moderate erosion in a limited area; score 3: more extended erosions; and score 4: general destruction. Neutrophil quantification was performed in three adjacent sections.

### Confocal microscopy

For immunostaining, 7-μm tissue sections of spleens were used. To analyze the populations of T helper 17 cells, we used PE-conjugated anti-CD4 and fluorescein isothiocyanate (FITC)-conjugated anti-IL-17 antibodies (eBioscience; San Diego, CA, USA). To analyze the populations of regulatory T cells, the samples were stained with FITC-conjugated anti-CD4, APC-conjugated anti-CD25, and PE-conjugated anti-Foxp3 antibodies. The stained sections were observed using a Zeiss microscope (LSM 510 Meta; Carl Zeiss, Oberkochen, Germany) at × 400 magnification. Positive cells were counted, and the values were expressed as the mean ± standard deviation.

### Flow cytometry

To quantify the populations of Th17 cells and Foxp3-positive Treg cells, splenocytes were stimulated with 25 ng/mL PMA and 250 ng/mL ionomycin (both from Sigma-Aldrich, St. Louis, MO, USA) and Golgi Stop (BD Biosciences, San Diego, CA, USA) in a 24-well plate and incubated for 4 h. The stimulated cells were stained with Percp-conjugated anti-CD4 antibody (eBiosciences), then fixed and permeabilized using the Cytofix/Cytoperm Plus kit (BD Biosciences), in accordance with the manufacturer’s instructions; they were then stained with FITC-conjugated anti-IL-17 antibody (eBioscience). For analysis of Treg cells, splenocytes were surface-labeled with anti-CD4 and anti-CD25 antibodies, followed by fixation, permeabilization, and intracellular staining with anti-Foxp3 antibody, in accordance with the manufacturer’s protocol. All samples were examined using a FACS Calibur device (BD Pharmingen).

### Murine T-cell isolation and differentiation

Spleen cell cultures were cultured in RPMI 1640 medium supplemented with 5% FBS. To purify CD4^+^ T cells, the cells were reacted with CD4-coated magnetic beads and isolated using magnetically activated cell sorting (MACS) separation columns (Miltenyi Biotec). Selected CD4 positive T cells were stimulated with plate-bound anti-CD3 (0.5 μg/mL), soluble anti-CD28 (1 μg/mL; both from BD Biosciences), anti-interferon-γ (2 μg/mL), anti-IL-4 (2 μg/mL) antibodies, recombinant TGF-β (2 ng/mL), and recombinant IL-6 (20 ng/mL) (R&D Systems) for 3 days to achieve polarization of Th17 cells.

### Co-culture of Th17 cells with adipocytes

Sorted Th17 cells were seeded in 24-well plates at 1 × 10^6^ per well in 5% FBS added-RPMI and brown/white adipocyte was a layer on to Th17 cells. Cells were incubated at 37 °C for 72 h. 3 days later, the culture supernatants were collected.

### Enzyme-linked immunosorbent assay (ELISA)

The quantity of IL-17 and IL-10 in culture supernatants were measured by sandwich EILSA (R&D Systems, Minneapolis, MN). Alkaline phosphatase (Sigma-Aldrich) was used for color development. The absorbance was determined at a wavelength of 405 nm on an ELISA microplate reader (Molecular Devices, Sunnyvale, CA, USA).

### Western blotting

Proteins were isolated by sodium dodecyl sulfate–polyacrylamide gel electrophoresis and transferred onto nitrocellulose membranes (Amersham Pharmacia Biotech, Piscataway, NJ, USA). Western blotting was assessed using a SNAP i.d. Protein Detection System (Millipore, Billerica, MA, USA). Membranes were stained with primary antibodies against Akt, p-Akt (Ser^473^), PI3K, p-PI3K and GAPDH (All from Cell Signaling, MA, USA).

### Transplantation of BAT

BAT was acquired from DBA1/J donor mice (age 7 weeks) and washed in sterile PBS. BAT of donor mice has coated with phenol red-free Matrigel (BD Biosciences). We then transplanted 0.2 g donor BAT into the subcutaneous dorsal region of CIA mice as quickly as possible. The spleen samples of recipient mice were obtained 5 weeks after the transplantation. The sham surgery group underwent the same procedure. The procedure was described in Fig. 1A.

### Microarray analysis

We performed microarray analysis using an Affymetrix Mouse Gene 2.0 ST array for 24,532 genes (Illumina) by Macrogen Co. (Seoul, Korea), in accordance with the manufacturer’s instructions.

### Statistical analysis

All data were analyzed using the nonparametric Mann–Whitney *U* test to compare two groups or one-way analysis of variance with Bonferroni’s post hoc test for multiple comparisons (≥ 3 groups). GraphPad Prism software (ver. 5.01) was employed for all analyses. A value of *P* < 0.05 was considered statistically significant. Data are expressed as means ± standard deviations.

## Supplementary information

Supplementary Information
