# Supplementary material for: Brown adipose tissue ameliorates autoimmune arthritis via inhibition of Th17 cells
Source: Sci Rep. 2020 Jul 23;10:12374. doi: 10.1038/s41598-020-68749-x (PMC7378076; doi:10.1038/s41598-020-68749-x)
Supplement: Supplementary file 1 — Supplementary Information [file 41598_2020_68749_MOESM1_ESM.docx]

**Brown adipose tissue ameliorates autoimmune arthritis via inhibition of Th17 cells**

Jeonghyeon Moon^1,6^*, Dasom Kim^2^*, Eun Kyung Kim^2^*, Seon-yeong Lee^2^, Hyun Sik Na^2^, Gyoung Nyun Kim^5^, Aram Lee^2^, KyungAh Jung^3^, Jeong Won Choi^2^, Sung-Hwan Park^2^, Sangho Roh^6^† and Mi-La Cho^1, 2^†

**Figure S1**


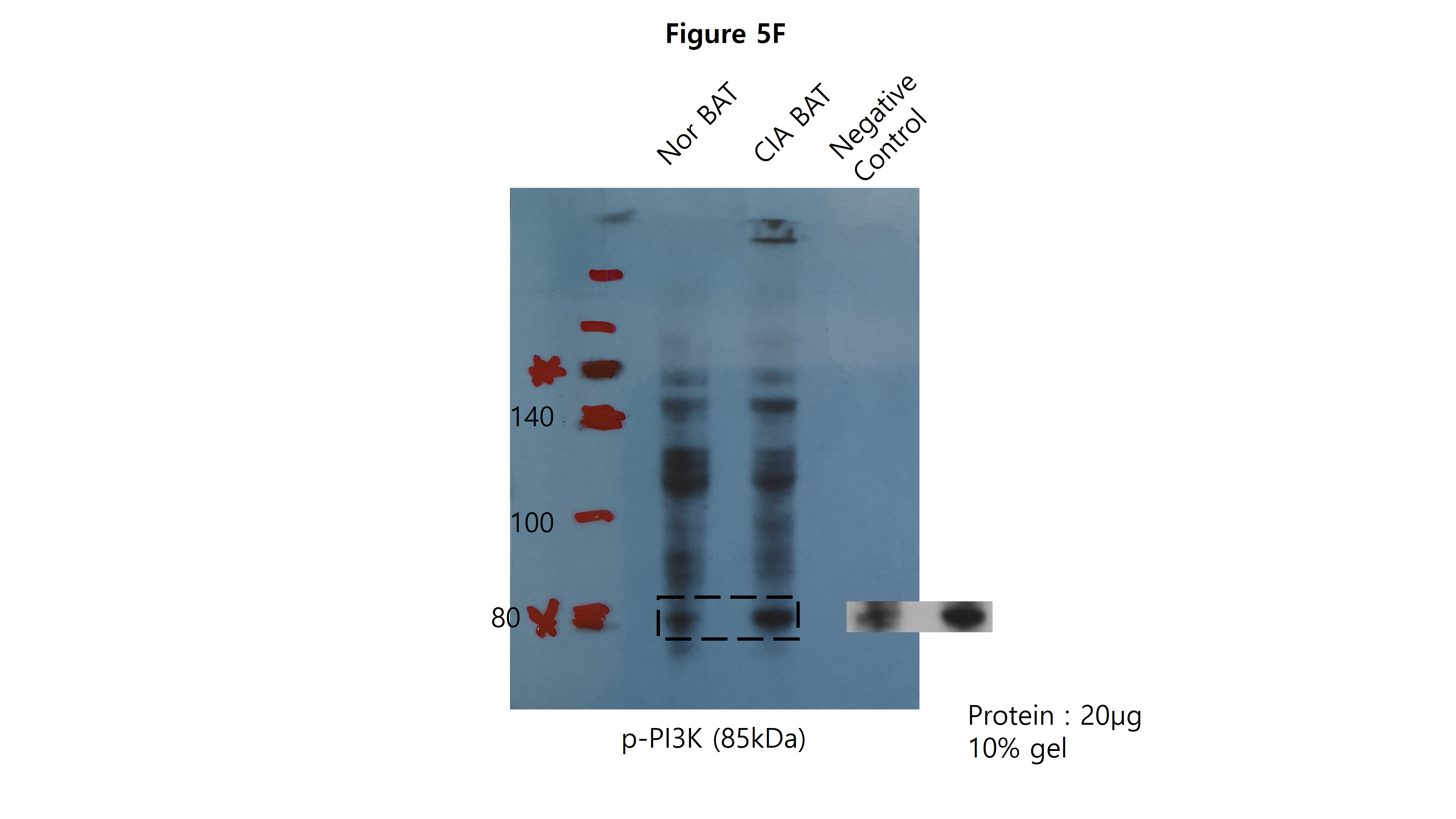

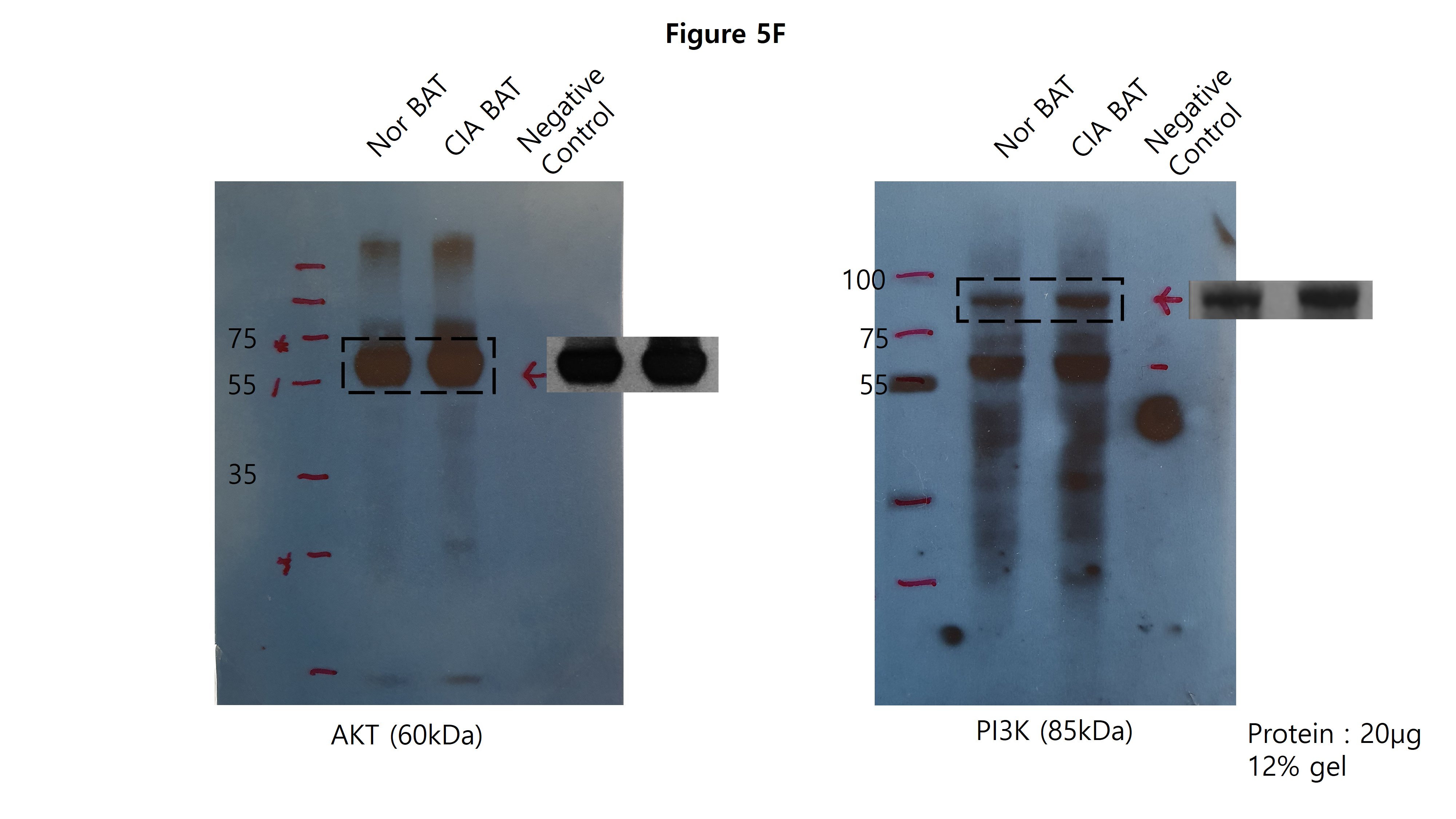

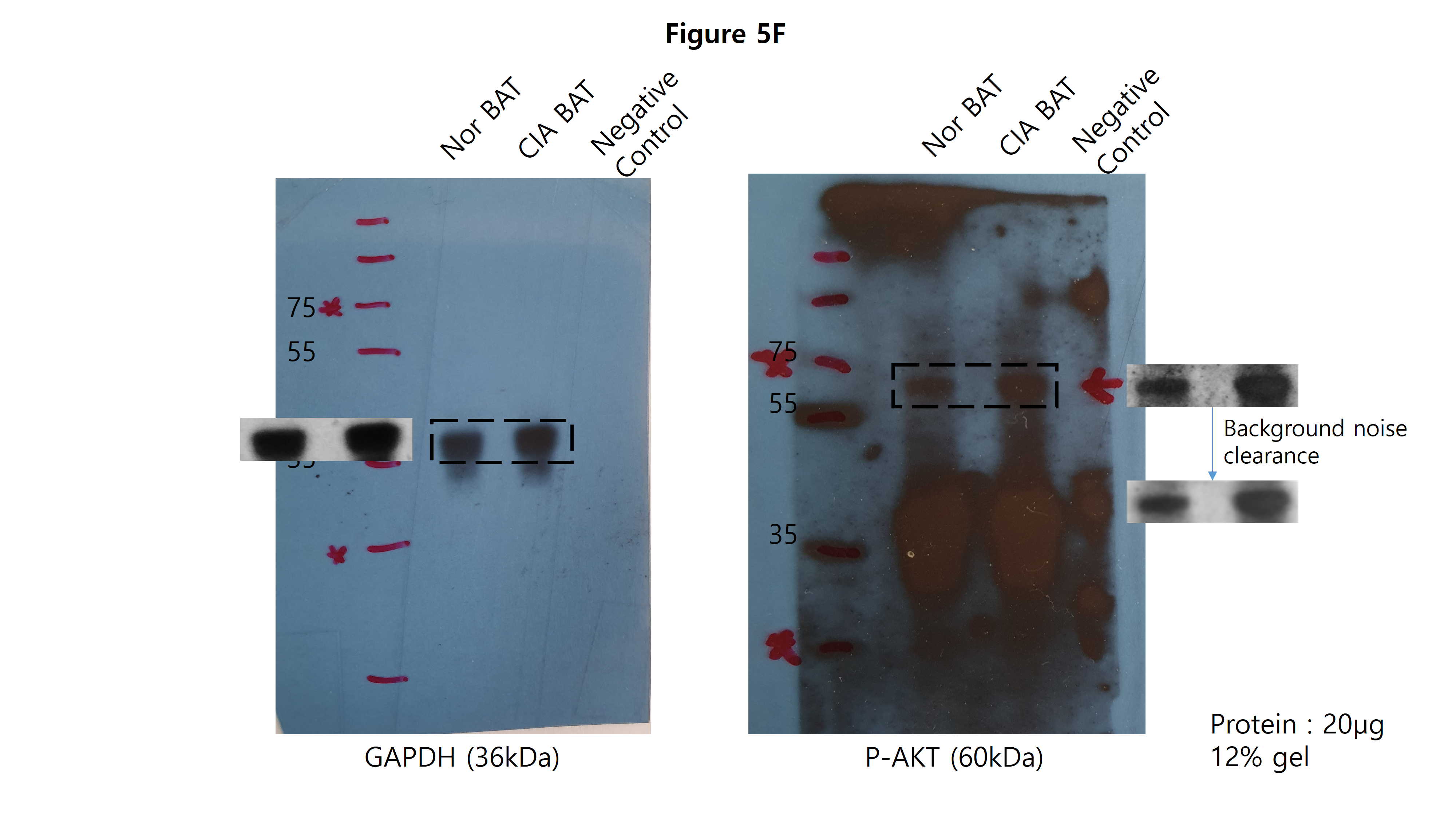


**Figure S1.** Gel data
